# Real-World Clinical Outcomes of Trilaciclib for the Prevention of Myelosuppression in Patients with Esophageal Cancer Undergoing Chemotherapy

**DOI:** 10.3390/curroncol32040189

**Published:** 2025-03-24

**Authors:** Hui Chen, Jingze Yan, Zeyuan Liu, Xiaolin Ge, Xinchen Sun, Xiaojie Xia

**Affiliations:** 1 Department of Radiation Oncology, The First Affiliated Hospital of Nanjing Medical University, Nanjing 210029, China; chenhui12342025@163.com (H.C.); yanjingze2025@163.com (J.Y.); doctorxlg@163.com (X.G.); 2 Department of Radiation Oncology, The Affiliated Jiangning Hospital of Nanjing Medical University, Nanjing 211199, China; dcliuzy@163.com; 3 Department of Oncology, Kangda College of Nanjing Medical University, Nanjing 210029, China

**Keywords:** trilaciclib, esophageal cancer, primary prevention, secondary prevention, myelosuppression

## Abstract

This study aims to evaluate the clinical effectiveness of trilaciclib in preventing myelosuppression in patients with esophageal cancer undergoing chemotherapy. Based on the use of trilaciclib, 81 patients were divided into a primary prevention group (PP group, n = 49) and a secondary prevention group (SP group, n = 32). The incidence of myelosuppression, antibiotic usage rate, survival outcomes, and other treatment-related toxicities were analyzed using chi-square tests and Kaplan–Meier survival curves. The incidence of chemotherapy-induced myelosuppression in the SP group was significantly higher than that in the PP group (96.9% vs. 79.6%), with a significantly higher proportion of grade III and above events (37.6% vs. 8.2%, *p* < 0.05). For chemotherapy-induced neutropenia, the incidence of grade III/IV events in the SP group was significantly higher than in the PP group (28.1% vs. 8.2%, *p* = 0.017). Additionally, the SP group experienced higher rates and severity of chemotherapy-induced anemia and thrombocytopenia. The PP group provided better protection against grade III/IV leukopenia and neutropenia (*p* < 0.05). Non-hematological toxicities and efficacy outcomes were similar between groups (*p* > 0.05). The study is the first to demonstrate that trilaciclib is a safe and effective option for the prevention of myelosuppression in esophageal cancer patients.

## 1. Introduction

Esophageal cancer (EC) is one of the deadliest cancers worldwide, primarily comprising esophageal squamous cell carcinoma (ESCC) and esophageal adenocarcinoma (EAC) [1]. ESCC is the most common type in Southeast Asia and Africa, and it occurs mostly in the middle and upper esophagus. It is a highly lethal cancer arising from the squamous epithelium of the esophagus. When exposed to carcinogens or subjected to long-term mechanical damage, esophageal epithelial cells undergo abnormal proliferation and eventually progress to invasive cancer [2]. And esophageal squamous cell carcinoma (ESCC) is the most common histological type in China, which is strongly linked to risk factors like smoking, heavy alcohol intake, and dietary patterns [3]. In recent years, advancements in immunotherapy and targeted therapies have significantly improved the treatment landscape for esophageal cancer. However, chemotherapy remains one of the cornerstone treatment modalities [4]. Although immune checkpoint inhibitors and targeted therapies have shown promise, their efficacy is often limited to specific molecular subtypes or advanced-stage patients, and not all patients respond equally to these treatments [5]. Chemotherapy, on the other hand, remains widely accessible, cost-effective, and effective across various disease stages, including resectable and metastatic cases [6,7]. Additionally, it is often used as the basis in combination therapies to enhance overall treatment efficacy and improve survival outcomes [8].

Myelosuppression is the most common adverse reaction during chemotherapy. According to the Report on the Current Status of Clinical Management of Chemotherapy-Induced Myelosuppression in China, the clinical incidence of chemotherapy-induced myelosuppression (CIM) in China is as high as 44.2% [9]. CIM primarily manifests as neutropenia, anemia, and thrombocytopenia, which often lead to reduced chemotherapy dosages, treatment delays, or even interruptions. These complications significantly compromise treatment efficacy and negatively affect patients’ quality of life [10]. Chemotherapy-induced myelosuppression occurs due to the cytotoxic effects of anticancer drugs on rapidly dividing hematopoietic progenitor cells in the bone marrow [11]. Since these cells are essential for blood cell production, their suppression results in diminished immune function, increased risk of infections, fatigue, and bleeding tendencies. The severity of CIM varies based on chemotherapy regimens, patient-specific factors such as age and comorbidities, and baseline bone marrow reserve [12]. Current clinical interventions for CIM mainly include the use of various hematopoietic growth factors and blood transfusions. However, these treatments typically target only specific blood cell lineages, require prolonged administration and repeatedly mobilize hematopoietic stem and progenitor cells (HSPCs). This repeated mobilization may result in bone marrow exhaustion and is often accompanied by adverse events such as bone pain, thrombosis, and fever [13].

Trilaciclib, a short-acting, selective, and reversible CDK4/6 inhibitor, is the first innovative drug globally approved for pan-lineage bone marrow protection. The development of CDK4/6 inhibitors in oncology has primarily focused on their role in tumor suppression by blocking cell cycle progression in cancer cells. However, the discovery of their ability to temporarily arrest hematopoietic stem and progenitor cells (HSPCs) in the G1 phase has opened a new avenue for their application in supportive cancer care [14,15]. As the first drug globally approved for bone marrow protection, trilaciclib has already been authorized in China for the prevention of chemotherapy-induced myelosuppression in patients receiving initial treatment for extensive-stage small-cell lung cancer (ES-SCLC) [16]. Clinical studies have demonstrated that trilaciclib effectively protects bone marrow in patients with small-cell lung cancer and triple-negative breast cancer [17,18,19]. Given the high hematologic toxicity associated with platinum- and taxane-based chemotherapy regimens in various malignancies, the potential benefits of trilaciclib extend beyond lung and breast cancer. For esophageal cancer patients undergoing chemotherapy, trilaciclib holds significant promise as an effective bone marrow protective strategy. However, clinical evidence for its use in this population remains lacking. This study is the first to explore the real-world clinical efficacy and safety of trilaciclib in the context of chemotherapy for esophageal cancer, providing evidence-based support for its application in this population.

## 2. Materials and Methods

### 2.1. Patients

This retrospective study included 81 patients with esophageal squamous cell carcinoma (ESCC) treated in the Radiation Oncology Department of Jiangsu Provincial People’s Hospital between December 2022 and October 2024. All patients received trilaciclib treatment. Inclusion criteria were: (1) age ≥ 18 years; (2) pathologically confirmed ESCC; (3) trilaciclib administration. Exclusion criteria were: (1) history of other malignancies; (2) difficulty in follow-up; (3) incomplete clinical data. Patient demographic and clinical baseline characteristics were collected retrospectively from electronic medical records. The extracted data included age, sex, body mass index (BMI), performance status (PS), smoking history, alcohol consumption history, tumor size, tumor location, and treatment regimen. Performance status was assessed according to the Eastern Cooperative Oncology Group (ECOG) scale. Clinical staging was based on the eighth edition of the Union for International Cancer Control (UICC) guidelines. Ethical approval was obtained from the Ethics Committee of Jiangsu Provincial People’s Hospital.

### 2.2. Treatment Regimens

Most patients underwent concurrent chemoradiotherapy, with or without targeted therapy and immunotherapy. A smaller proportion received chemotherapy and immunotherapy. Radiotherapy was delivered using intensity-modulated radiation therapy (IMRT) at a total dose of 50–50.4 Gy (1.8–2.0 Gy per fraction). The chemotherapy regimen consisted of paclitaxel (135 mg/m^2^ on day 1) and nedaplatin (75 mg/m^2^ on days 1–2), administered every three weeks (Q3W). Immunotherapy involved anti-PD-1 antibodies, administered with Q3W. Targeted therapy consisted of anlotinib (10–12 mg once daily, days 1–14). Individualized treatment plans were selected based on the patient’s condition and preferences. Trilaciclib (240 mg/m^2^) was administered via intravenous injection 4 h before chemotherapy. Patients were divided into two groups based on the timing of trilaciclib administration: the primary prevention (PP) group and the secondary prevention (SP) group. Patients in the PP group received trilaciclib pre-chemotherapy starting from the first cycle. Patients in the SP group initiated trilaciclib only after experiencing myelosuppression in prior chemotherapy cycles. For those experiencing myelosuppression during treatment, granulocyte colony-stimulating factors (G-CSFs), either long-acting or short-acting, were administered following the NCCN Hematopoietic Growth Factors Guidelines.

### 2.3. Study Endpoints

The primary endpoint was the incidence of myelosuppression during treatment, including leukopenia, neutropenia, anemia, and thrombocytopenia. Key secondary endpoints included febrile neutropenia (FN) and other systemic adverse events. FN was defined as a single oral temperature > 38.3 °C or a sustained temperature > 38.0 °C for over 1 h, accompanied by a neutrophil count < 500 cells/μL. The assessment of myelosuppression and other adverse events followed the Common Terminology Criteria for Adverse Events (CTCAE, version 5.0). Additional endpoints included antibiotic use, tumor response, and overall survival (OS). Tumor response was evaluated according to the Response Evaluation Criteria in Solid Tumors (RECIST 1.1) and classified as complete response (CR), partial response (PR), stable disease (SD), or progressive disease (PD). Objective response rate (ORR) was defined as CR + PR, while disease control rate (DCR) was defined as CR + PR + SD. All patients underwent at least two chemotherapy cycles, with short-term efficacy assessed via imaging (CT or MRI).

### 2.4. Statistical Analysis

All statistical analyses were performed using SPSS version 22.0 (IBM Corp., Armonk, NY, USA). Patient characteristics, adverse events, and tumor responses were analyzed using Pearson’s chi-square test or Fisher’s exact test. Survival curves were generated using the Kaplan–Meier method and compared with the log-rank test. All statistical tests were two-sided, with a *p*-value < 0.05 considered statistically significant.

### 2.5. Ethics Statement

The study was conducted in accordance with the Declaration of Helsinki. The Ethics Committee of the First Affiliated Hospital of Nanjing Medical University approved the study protocol and the registration number is 2024-SR-313.

## 3. Results

### 3.1. Patient Characteristics

A total of 81 patients were included (PP group: 49, SP group: 32). Table 1 shows no statistically significant differences in baseline characteristics between the PP and SP groups, including age, sex, BMI, PS score, nutritional status, smoking and alcohol history, tumor stage, differentiation, treatment regimens, and tumor length (*p* > 0.05).

### 3.2. Hematological Toxicity

**Figure 1:**
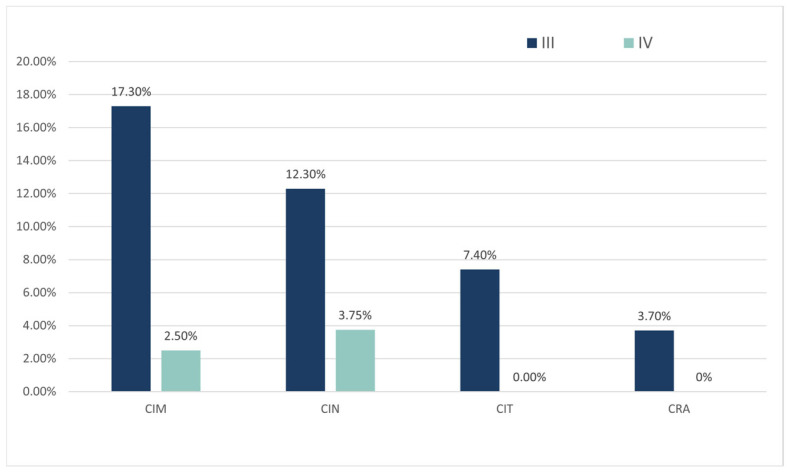
Incidence rate of grade III and grade IV bone marrow suppression. CIM: chemotherapy-induced myelosuppression; CIN: chemotherapy-induced neutropenia; CIT: chemotherapy-induced thrombocytopenia; CRA: chemotherapy-related anemia.

As shown in Table 2, the overall incidence of CIM was 86.4%, predominantly grade I (25.9%) and grade II (40.7%), with a lower incidence of grade III or higher events (19.8%). The incidence of chemotherapy-induced neutropenia (CIN) was 59.3%, primarily grade II (27.2%). The incidence of chemotherapy-induced thrombocytopenia (CIT) was 50.6%, with grade I (27.2%) being the most common. Chemotherapy-induced anemia (CRA) had the highest incidence (69.1%), dominated by grade I events (46.9%). Figure 1 indicates that grade III events occurred more frequently than grade IV across all types of hematological toxicity (CIM: grade III 17.3% vs. grade IV 2.5%; CIN: grade III 12.3% vs. grade IV 3.7%; CIT: grade III 7.4% vs. grade IV 0%; CRA: grade III 3.7% vs. grade IV 0%).

**Figure 2:**
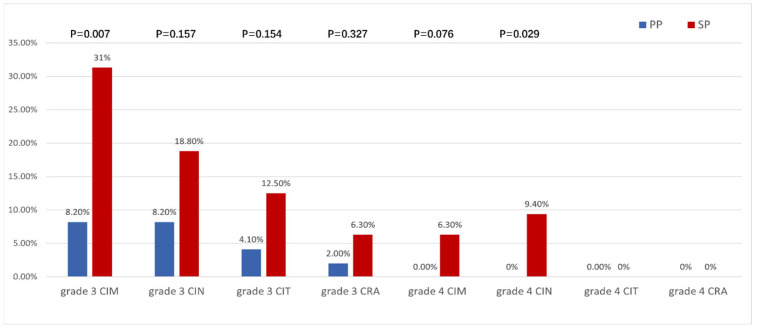
Incidence of grade III and IV bone marrow suppression events in the PP group and SP group. CIM: chemotherapy-induced myelosuppression; CIN: chemotherapy-induced neutropenia; CIT: chemotherapy-induced thrombocytopenia; CRA: chemotherapy-related anemia; PP: primary prevention; SP: secondary prevention.

Table 3 compares the incidence and severity of myelosuppression between the PP and SP groups. The incidence of CIM was significantly higher in the SP group than in the PP group (96.9% vs. 79.6%), with higher rates of grade III/IV events (31.3% vs. 8.2%; 6.3% vs. 0%, respectively). Similarly, the overall incidence of CIN was higher in the SP group compared to the PP group (71.9% vs. 51.0%), with significantly more grade III/IV events in the SP group (18.8% vs. 8.2%; 9.4% vs. 0%). For CIT, the total incidence was also higher in the SP group (65.6% vs. 40.8%), particularly for grade II and III events (31.3% and 12.5% in the SP group vs. 6.1% and 4.1% in the PP group). CRA was more common in the SP group (81.3% vs. 61.2%), with a higher incidence of grade III events (6.3% vs. 2.0%). These results demonstrate that the PP group had significantly lower rates and severity of CIM, CIN, CIT, and CRA compared to the SP group. Figure 2 highlights the differences in the incidence of grade III/IV myelosuppression between the two groups, with the SP group showing higher severity across all types.

**Figure 3:**
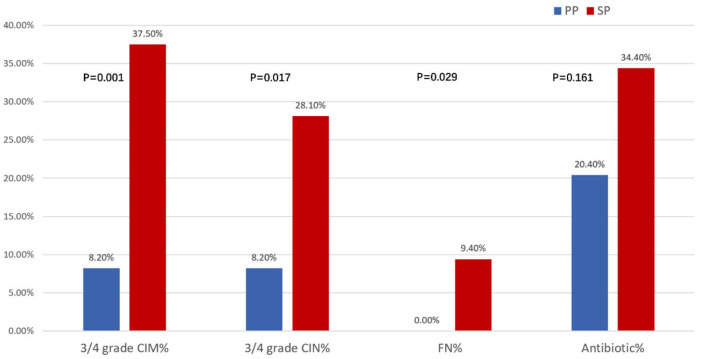
Myeloprotection outcomes associated with PP and SP groups. CIM: chemotherapy-induced myelosuppression; CIN: chemotherapy-induced neutropenia; FN: febrile neutropenia.

As shown in Figure 3, the PP group had significantly lower incidences of grade III/IV leukopenia and neutropenia compared to the SP group (CIM: 8.2% vs. 37.5%, *p* = 0.001; CIN: 8.2% vs. 28.1%, *p* = 0.017). Antibiotic use was lower in the PP group than in the SP group (20.4% vs. 34.4%, *p* > 0.05). Notably, no patients in the PP group experienced febrile neutropenia (FN), while three cases were reported in the SP group. Overall, primary prophylaxis with trilaciclib provided superior protection against leukopenia, neutropenia, anemia, and thrombocytopenia compared to secondary prophylaxis in esophageal cancer patients receiving chemotherapy.

### 3.3. Safety

Non-hematological toxicities, summarized in Table 4, included abnormal liver enzymes, cough, fatigue, nausea, diarrhea, constipation, radiation pneumonitis, radiation dermatitis, and electrolyte disturbances. There were no significant differences in the incidence of non-hematological toxicities between the PP and SP groups (*p* > 0.05).

### 3.4. Efficacy and Survival Outcomes

**Figure 4:**
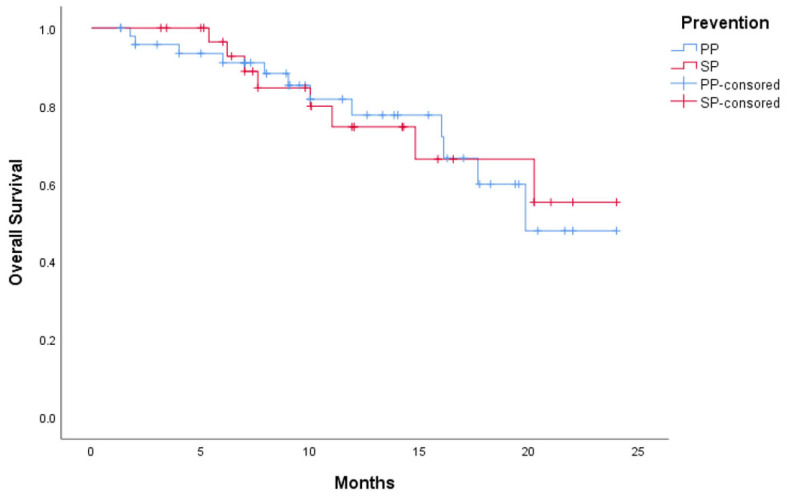
Kaplan–Meier curves for the PP and SP groups.

As shown in Table 5, in the PP group, 22 patients exhibited a complete response (CR), 23 a partial response (PR), 2 stable disease (SD), and 2 progressive disease (PD). In the SP group, 13 patients achieved CR, 14 PR, 3 SD, and 2 PD. The overall response rates (ORR) were similar between the PP and SP groups (91.8% vs. 84.4%), as were disease control rates (DCR: 95.9% vs. 93.8%), with no statistically significant differences. The 1-year overall survival (OS) rates were also comparable (PP: 83.7% vs. SP: 81.3%, *p* > 0.05; Figure 4).

## 4. Discussion

Esophageal cancer ranks as the seventh most common malignancy and the sixth leading cause of cancer-related deaths globally [20]. Surgery, radiotherapy, chemotherapy, immunotherapy, and targeted therapy represent the primary treatment modalities for esophageal cancer, which are often used in combination depending on the stage of disease. Among these, chemotherapy remains the cornerstone of treatment [21].

Chemotherapy-induced myelosuppression (CIM) is one of the most common hematologic toxicities during chemotherapy. Over 80% of chemotherapy agents can induce CIM through mechanisms such as promoting hematopoietic stem cell senescence, apoptosis, or damaging the hematopoietic microenvironment [22]. CIM primarily manifests as neutropenia, anemia, and thrombocytopenia. Severe cases can lead to febrile neutropenia (FN), a life-threatening complication. Research has shown that FN increases early mortality rates by 15% and reduces overall survival by 48%. Similarly, chemotherapy-induced thrombocytopenia (CIT) heightens the risk of bleeding, which in severe cases may become life-threatening. Chemotherapy-induced anemia (CIA) can cause systemic ischemic and hypoxic damage, accelerating disease progression and negatively affecting prognosis [10]. CIM significantly affects cancer patients, often leading to reduced chemotherapy doses, delayed treatment schedules, and prolonged hospitalizations, ultimately compromising treatment efficacy and continuity [14,23,24]. Traditional approaches to managing CIM, such as prolonged hospitalization or additional supportive treatments, often fail to provide true bone marrow protection and may exacerbate complications, including the risk of bone marrow exhaustion [25].

Trilaciclib, a highly selective, reversible CDK4/6 inhibitor, is administered intravenously before chemotherapy to protect hematopoietic stem and progenitor cells (HSPCs) and improve antitumor efficacy [26]. This study analyzed the clinical effects of trilaciclib in preventing CIM among 81 esophageal cancer patients, including 49 in the primary prophylaxis (PP) group and 32 in the secondary prophylaxis (SP) group. The results demonstrated that the PP group experienced a significantly lower incidence of CIM (79.6% vs. 96.9%) compared to the SP group, especially for grade III/IV events (8.2% vs. 31.3%). No grade IV CIM events were observed in the PP group. These findings suggest that primary prophylaxis is more effective than secondary prophylaxis in reducing the incidence and severity of CIM, particularly in preventing grade III/IV events. This advantage may be attributed to the early initiation of prophylactic intervention before the first cycle of chemotherapy, which prevents chemotherapy-induced bone marrow damage and dysfunction at an early stage. Specifically, trilaciclib protects HSPCs by arresting them in the G1 phase, thereby reducing their susceptibility to chemotherapy-induced damage. After chemotherapy, these cells resume maturation and differentiation into myeloid cells, lymphocytes, and other hematopoietic lineages. Preclinical studies further support this mechanism. For example, an in vitro study using HS68 cells (a CDK4/6-dependent HSPC model) demonstrated that trilaciclib protects HSPCs from chemotherapy-induced apoptosis and DNA damage. Similarly, studies in FVB/N mouse models showed that trilaciclib reduced chemotherapy-induced HSPC damage, accelerated post-chemotherapy hematologic recovery, and provided protection across all hematopoietic lineages [15]. Additionally, trilaciclib has been shown to enhance immune response by protecting lymphocytes and promoting T-cell proliferation, thereby improving cellular immunity [17]. In this study, the PP group had a lower rate of antibiotic use compared to the SP group (20.4% vs. 34.4%), and no FN events were observed in the PP group, whereas three cases occurred in the SP group. This suggests that primary prophylaxis not only provides superior protection against CIM, but also reduces the risk of chemotherapy-associated infections. Furthermore, there was no significant difference between the two groups in non-hematologic toxicities, such as liver enzyme abnormalities, gastrointestinal symptoms, electrolyte disturbances, or radiation-related complications, indicating that primary prophylaxis did not increase the burden of non-hematologic adverse events and was well tolerated. Basic research has demonstrated that in tumor mouse models, whether CDK4/6-dependent or independent, trilaciclib does not interfere with the antitumor activity of chemotherapy [17,27,28]. Similarly, the G1T28-04 clinical trial in triple-negative breast cancer revealed that trilaciclib did not compromise chemotherapy efficacy, regardless of CDK4/6 dependency [29]. Consistent with these findings, our study demonstrated comparable overall response rates (ORR), disease control rates (DCR), and one-year overall survival (OS) between the PP and SP groups (ORR: 91.8% vs. 84.4%; DCR: 95.9% vs. 93.8%; one-year OS: 83.7% vs. 81.3%). This confirms that trilaciclib does not adversely affect the antitumor efficacy of chemotherapy while significantly improving CIM outcomes.

This study has several limitations. First, as a retrospective analysis, potential selection bias may exist. Large-scale prospective randomized controlled trials are warranted to validate these findings. Second, the sample size was limited due to trilaciclib’s recent market introduction and high cost, which may affect generalizability. Lastly, long-term follow-up data are lacking; extended observation is needed to comprehensively evaluate trilaciclib’s safety and efficacy.

Most clinical studies of trilaciclib to date have focused on patients with small cell lung cancer (SCLC) and triple-negative breast cancer. Three randomized, double-blind phase II trials in SCLC patients (G1T28-02, G1T28-05, and G1T28-03) demonstrated that trilaciclib significantly reduced the duration of severe neutropenia during the first chemotherapy cycle (from four days to zero days) and decreased the incidence of grade III/IV neutropenia, anemia, and thrombocytopenia by 78.4%, 36.3%, and 45.9%, respectively (all *p* < 0.05) [19]. These findings led to trilaciclib being recognized as a breakthrough therapy in the United States in 2019 and its subsequent approval for use in extensive-stage SCLC patients undergoing chemotherapy. Trilaciclib was approved for use in China in 2022. However, no clinical data have previously been available regarding its use in esophageal cancer [30]. Our study fills this gap, providing valuable insights into its potential application in this patient population.

## 5. Conclusions

The study is the first to demonstrate that the use of trilaciclib before chemotherapy effectively reduced myelosuppression in esophageal cancer patients, with better outcomes in the primary prevention group. Trilaciclib is a safe and effective option for the prevention of myelosuppression in esophageal cancer patients.

## Figures and Tables

**Table 1:** Baseline information of patients before and after grouping.

		Total (n = 81)	PP (n = 49)	SP (n = 32)	*p*
Gender	Male	65 (80.2%)	40 (81.63%)	25 (78.13%)	0.698
	Female	16 (19.8%)	9 (18.37%)	7 (21.88%)	
Age	<65	19 (23.5%)	11 (22.45%)	8 (25.00%)	0.791
	≥65	62 (76.5%)	38 (77.55%)	24 (75.00%)	
BMI	<18.5	13 (16.0%)	8 (16.33%)	5 (15.63%)	0.98
	18.5–24	47 (58.0%)	28 (57.14%)	19 (59.38%)	
	≥24	21 (26.0%)	13 (26.53%)	8 (25.00%)	
Smoking	Yes	35 (43.2%)	23 (46.94%)	12 (37.50%)	0.402
	No	46 (56.8%)	26 (53.06%)	20 (62.50%)	
Alcohol abuse	Yes	38 (46.9%)	25 (51.02%)	13 (40.63%)	0.359
	No	43 (53.1%)	24 (48.98%)	19 (59.38%)	
Nutritional management mode	Soft diet	19 (23.5%)	11 (22.45%)	8 (25.00%)	0.897
	Semi-liquid diet	41 (50.6%)	24 (48.98%)	17 (53.13%)	
	Liquid diet	17 (21.0%)	11 (22.45%)	6 (18.75%)	
	Naso-intestinal tube/gastrostomy tube	4 (4.9%)	3 (6.12%)	1 (3.13%)	
PS score	0	17 (21.0%)	13 (26.53%)	4 (12.50%)	0.13
	1	64 (79.0%)	36 (73.47%)	28 (87.50%)	
Clinical stage	I	3 (3.7%)	3 (6.12%)	0 (0.00%)	0.502
	II	19 (23.5%)	11 (22.45%)	8 (25.00%)	
	III	24 (29.6%)	15 (30.61%)	9 (28.13%)	
	IV	35 (43.2%)	20 (40.82%)	15 (46.88%)	
Grade	G1	4 (4.9%)	1 (2.04%)	3 (9.38%)	0.242
	G2	38 (46.9%)	22 (44.90%)	16 (50.00%)	
	G3	39 (48.1%)	26 (53.06%)	13 (40.63%)	
Tumor size	<5 cm	25 (30.9%)	15 (30.61%)	10 (31.25%)	0.952
	≥5 cm	56 (69.1%)	34 (69.39%)	22 (68.75%)	
Tumor location	Cervical	11 (13.6%)	8 (16.33%)	3 (9.38%)	0.819
	Upper chest	16 (19.8%)	10 (20.41%)	6 (18.75%)	
	Middle chest	16 (19.8%)	9 (18.37%)	7 (21.88%)	
	Lower chest	17 (21.0%)	11 (22.45%)	6 (18.75%)	
	Overlapping	21 (26.0%)	11 (22.45%)	10 (31.25%)	
Therapy	RT + CT	12 (14.8)	6 (12.24%)	6 (18.75%)	0.319
	RT + CT + TT	1 (1.2%)	1 (2.04%)	0 (0.00%)	
	RT + CT + IT	59 (72.8%)	36 (73.47%)	23 (71.88%)	
	RT + CT + IT + TT	5 (6.2%)	2 (4.08%)	3 (9.38%)	
	CT + IT	4 (4.9%)	4 (8.16%)	0 (0.00%)	
Prevention	Primary	49 (60.5%)	49 (100.00%)	0 (0.00%)	1.00
	Secondary	32 (39.5%)	0 (0.00%)	32 (100.00%)	

PP: primary prevention; SP: secondary prevention; PS: performance status; CRT: chemoradiotherapy; BMI: body mass index (calculated as weight in kilograms divided by height in meters squared); RT: radiotherapy; CT: chemotherapy; TT: targeted therapy; IT: immunotherapy.

**Table 2:** Percentage of various bone marrow suppression events in patients.

CIM	%	CIN	%	CIT	%	CRA	%
I	21 (25.9%)	I	13 (16.0%)	I	22 (27.2%)	I	38 (46.9%)
II	33 (40.7%)	II	22 (27.2%)	II	13 (16.0%)	II	15 (18.5%)
III	14 (17.3%)	III	10 (12.3%)	III	6 (7.4%)	III	3 (3.7%)
IV	2 (2.5%)	IV	3 (3.7%)	IV	0 (0%)	IV	0 (0%)
ALL	70 (86.4%)	ALL	48 (59.3%)	ALL	41 (50.6%)	ALL	56 (69.1%)

CIM: chemotherapy-induced myelosuppression; CIN: chemotherapy-induced neutropenia; CIT: chemotherapy-induced thrombocytopenia; CRA: chemotherapy-related anemia.

**Table 3:** Percentage of various bone marrow suppression events in patients in the PP group and SP group.

	CIM		CIN		CIT		CRA	
	PP	SP	PP	SP	PP	SP	PP	SP
I	17 (34.7%)	4 (12.5%)	8 (16.3%)	5 (15.6%)	15 (30.6%)	7 (21.9%)	21 (42.9%)	17 (53.1)
II	18 (36.7%)	15 (46.9%)	13 (26.5%)	9 (28.1%)	3 (6.1%)	10 (31.3%)	8 (16.3%)	7 (21.9%)
III	4 (8.2%)	10 (31.3%)	4 (8.2%)	6 (18.8%)	2 (4.1%)	4 (12.5%)	1 (2.0%)	2 (6.3%)
IV	0 (0%)	2 (6.3%)	0 (0%)	3 (9.4%)	0 (0%)	0 (0%)	0 (0%)	0 (0%)
ALL	39 (79.6%)	31 (96.9%)	25 (51.0%)	23 (71.9%)	20 (40.8%)	21 (65.6%)	30 (61.2%)	26 (81.3%)

PP: primary prevention; SP: secondary prevention; CIM: chemotherapy-induced myelosuppression; CIN: chemotherapy-induced neutropenia; CIT: chemotherapy-induced thrombocytopenia; CRA: chemotherapy-related anemia.

**Table 4:** The remaining adverse reactions in the PP group and SP group.

TRAE	PP (n = 49)				SP (n = 32)				*p*-Value
	0–1	2	3	4	0–1	2	3	4	
Fatigue	42 (85.7%)	7 (14.3%)	0	0	31 (96.9%)	1 (3.1%)	0	0	0.182
Decreased appetite	41 (83.7%)	8 (16.3%)	0	0	28 (87.5%)	2 (6.3%)	2 (6.3%)	0	0.134
Hepatic function abnormal	43 (87.8%)	3 (6.1%)	3 (6.1%)	0	26 (81.3%)	4 (12.5%)	2 (6.3%)	0	0.793
Renal function abnormal	48 (97.9%)	1 (2.1%)	0	0	31 (96.9%)	1 (3.1%)	0	0	0.401
Vomiting	46 (93.9%)	3 (6.1%)	0	0	32 (100%)	0	0	0	0.06
Hiccups	49 (100%)	0	0	0	32 (100%)	0	0	0	0.645
Stomatitis	40 (81.6%)	9 (18.4%)	0	0	28 (87.5%)	4 (12.5%)	0	0	0.554
Cough	49 (100%)	0	0	0	31 (96.9%)	1 (3.1%)	0	0	0.245
Constipation	49 (100%)	0	0	0	32 (100%)	0	0	0	0.129
Diarrhea	49 (100%)	0	0	0	31 (96.9%)	1 (3.1%)	0	0	0.414
Radiation dermatitis	48 (97.9%)	0	1 (2.1%)	0	31 (96.9%)	1 (3.1%)	0	0	0.209
Radiation pneumonitis	48 (97.9%)	1 (2.1%)	0	0	32 (100%)	0	0	0	0.589
Hypocalcemia	47 (96.0%)	1 (2.1%)	1 (2.1%)	0	29 (90.6%)	3 (9.4%)	0	0	0.053
Hyponatremia	47 (96.0%)	2 (4.1%)	0	0	31 (96.9%)	1 (3.1%)	0	0	0.926
Hyperkalemia	47 (96.0%)	2 (4.1%)	0	0	32 (100%)	0	0	0	0.492
Hypokalemia	46 (93.9%)	3 (6.1%)	0	0	31 (96.9%)	0	0	0	0.662

TRAE: treatment-related adverse event.

**Table 5:** Comparison of efficacy between the PP group and the SP group.

	PP Group	SP Group	*p*-Value
CR	22 (44.9%)	13 (40.6%)	0.704
PR	23 (46.9%)	14 (43.8%)	0.778
SD	2 (4.1%)	3 (9.4%)	0.333
PD	2 (4.1%)	2 (6.3%)	0.66
ORR	91.80%	84.40%	0.161
DCR	95.90%	93.80%	0.66

CR: complete response; PR: partial response; SD: stable disease; PD: progressive disease; ORR: objective response rate; DCR: disease control rate.

## Data Availability

Data are available from the corresponding author upon reasonable request.

## Abbreviations

The following abbreviations are used in this manuscript:

CIA	Chemotherapy-induced anemia
CIM	Chemotherapy-induced myelosuppression
CIN	Chemotherapy-induced neutropenia
CIT	Chemotherapy-induced thrombocytopenia
CR	Complete response
CRA	Chemotherapy-induced Anemia
CTCAE	Common Terminology Criteria for Adverse Events
DCR	Disease control rate
ESCC	Esophageal squamous cell carcinoma
ES-SCLC	Extensive-stage small-cell lung cancer
FN	Febrile neutropenia
G-CSFs	Granulocyte colony-stimulating factors
HSPCs	Hematopoietic stem and progenitor cells
IMRT	Intensity-modulated radiation therapy
ORR	Objective response rate
OS	Overall survival
PD	Progressive disease
PP	Primary prevention
PR	Partial response
Q3W	Every three weeks
RECIST	Response Evaluation Criteria in Solid Tumors
SCLC	Small-cell lung cancer
SD	Stable disease
SP	Secondary prevention
UICC	Union for International Cancer Control
